# Reshaping human neurons

**DOI:** 10.7554/eLife.110981

**Published:** 2026-03-31

**Authors:** Jessica L Maltman, Javier González-Maeso

**Affiliations:** 1 Biomedical Sciences Graduate Program, Virginia Commonwealth University School of Medicine Richmond United States; 2 Department of Pharmacology and Toxicology, Virginia Commonwealth University School of Medicine Richmond United States

**Keywords:** psychedelics, neuroplasticity, human induced pluripotent stem cell derived cortical neurons, 5-HT2A receptor, psilocin, BDNF, Human

## Abstract

Human neurons derived from stem cells show increased structural complexity and stronger synaptic connections after exposure to psilocin, the active metabolite of the psychedelic psilocybin.

**Related research article** Schmidt M, Hoffrichter A, Davoudi M, Horschitz S, Lau T, Meinhardt M, Spanagel R, Ladewig J, Köhr P, Koch G. 2026. Psilocin fosters neuroplasticity in iPSC-derived human cortical neurons. *eLife* **14**:RP104006. doi: 10.7554/eLife.104006.

The adult brain is not static. Neurons can change their structure and connections in response to experience, learning and certain drugs. This property, known as neuroplasticity, allows the brain to adapt throughout life. Increasing evidence suggests that plasticity plays a key role in treatments for several psychiatric disorders, including depression and anxiety (Duman and Aghajanian, 2012).

In recent years, classical psychedelics such as psilocybin (found in so-called magic mushrooms) and lysergic acid diethylamide (more commonly known as LSD) have attracted renewed interest in neuroscience and molecular psychiatry (Kwan et al., 2022). These compounds alter perception and cognition mainly by activating serotonin receptors in the brain. Clinical studies also suggest that psychedelic treatments can produce rapid and lasting improvements in some patients with depression and other psychiatric conditions (Jacobs et al., 2026). This is striking because conventional antidepressants, such as fluoxetine, often require weeks of repeated treatment before benefits appear. Increasing evidence suggests that psychedelics may produce these effects by temporarily enhancing neuroplasticity, allowing the brain to reorganize its circuits.

Most of what we know about the molecular and cellular mechanisms underlying psychedelic-induced neuroplasticity comes from studies in rodents (Ly et al., 2018). Now, in eLife, Philipp Koch and colleagues – including Malin Schmidt as first author – examine this process in human neurons grown in cell cultures (Schmidt et al., 2026). Their work provides important clues about the mechanisms through which psychedelics reshape human brain cells and helps bridge the gap between preclinical animal studies and human biology.

The researchers – who are based at the University of Heidelberg/Medical Faculty Mannheim, the Hector Institute for Translational Brain Research, the German Cancer Research Center, and other institutes in Germany – used neurons derived from human induced pluripotent stem cells (iPSCs). These cells are created by reprogramming adult cells, such as skin fibroblasts, into a stem-cell-like state that can then be differentiated into different types of neurons in cell culture (Takahashi et al., 2007). Schmidt et al. generated cortical neurons resembling those found in the human frontal cortex, a region of the brain that is important for cognition, emotional regulation and decision-making.

The neurons were exposed to psilocin, the active metabolite produced in the body after psilocybin is consumed. The researchers then measured several indicators of neuroplasticity, including changes in neuronal structure, gene expression, synaptic proteins and electrophysiological activity. Even brief exposure to psilocin produced measurable changes (Figure 1). Within about 24 hours, the treated neurons developed more complex branching structures. These structures, called neurites, form the physical framework through which neurons connect (Yuste and Bonhoeffer, 2001). Greater branching increases opportunities for communication between cells and may support processes such as learning, sensory gating and memory.

**Figure 1.**
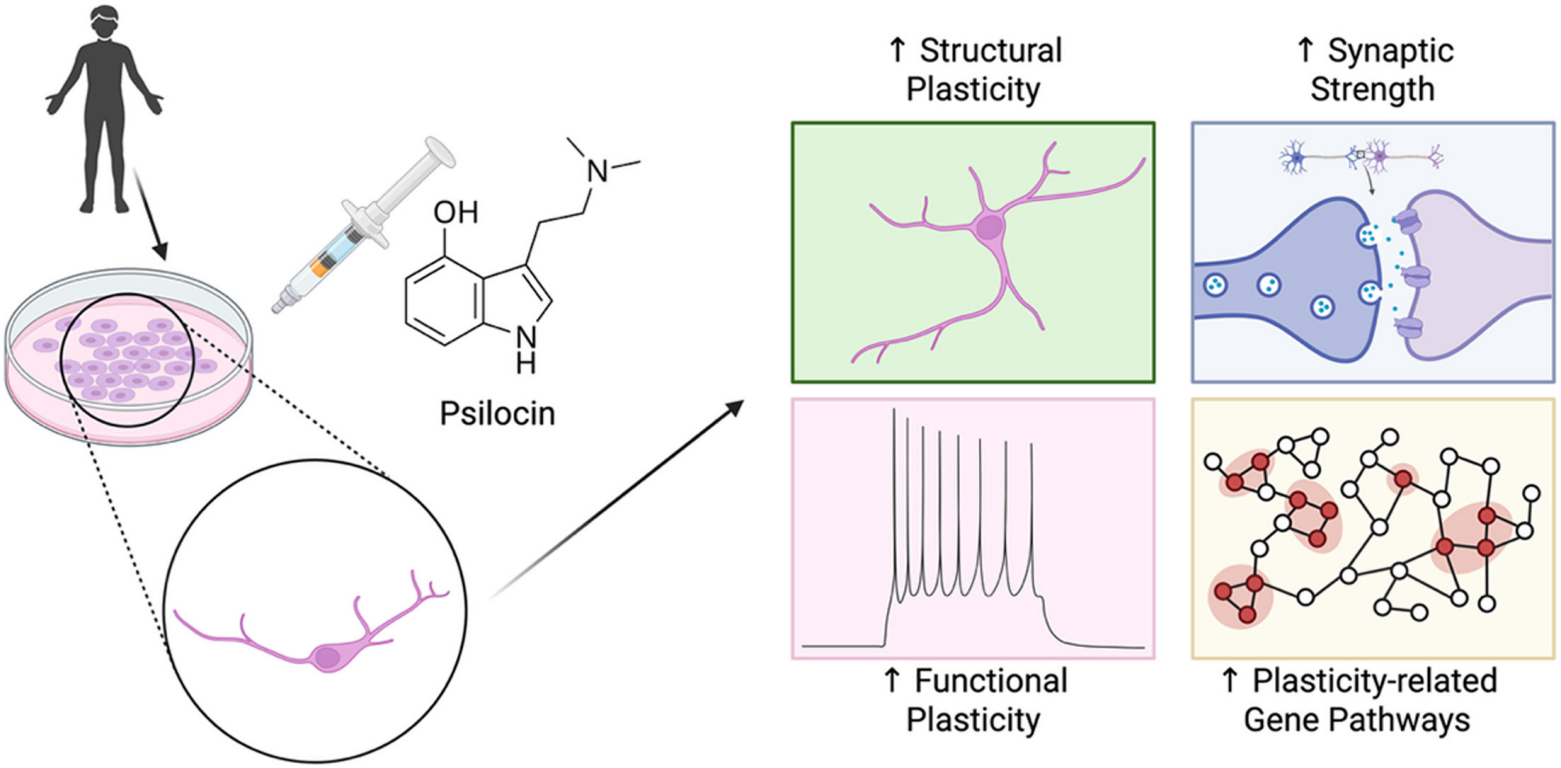
Psilocin promotes multiple forms of neuroplasticity in human cortical neurons. Human induced pluripotent stem cells (iPSCs) generated from adult tissue can be differentiated into cortical neurons in culture. Exposure to psilocin, the active metabolite of the classical psychedelic psilocybin, increases structural complexity, strengthens synaptic connections, enhances neuronal activity, and alters gene pathways associated with neuronal plasticity.

Psilocin also strengthened synaptic connections. The researchers observed increased levels of proteins that mark the pre- and postsynaptic sides of synapses. These proteins help organize the molecular machinery that allows neurons to release and detect neurotransmitters. When these structures become larger or more closely aligned, communication between neurons generally becomes more efficient. The functional consequences of these structural changes appeared days later. After about a week of continuous psilocin exposure, neurons showed stronger electrical activity, firing more action potentials and producing larger excitatory signals between cells. Together, these findings suggest that psilocin enhances both the physical and functional connectivity of neuronal networks.

The study further sheds light on the molecular pathways involved. Many of the observed effects depended on activation of the serotonin 5-HT_2A_ receptor, a G protein-coupled receptor widely recognized as the primary target of classical psychedelics (González-Maeso et al., 2007). Pharmacological blockade of this receptor with ketanserin (a serotonin 5-HT_2A_ receptor antagonist) prevented several of the plasticity-related changes. Psilocin altered the expression of genes linked to neuronal growth and synaptic remodeling, including immediate early genes that regulate long-term plasticity and learning. Schmidt et al. further observed increased levels of brain-derived neurotrophic factor (BDNF), a signaling molecule that promotes neuronal growth and is strongly linked to mood regulation (Wang et al., 2022). Interestingly, this increase in BDNF required both receptor signaling and receptor internalization via endocytosis. This finding suggests that psychedelic signaling may persist within intracellular compartments after receptor trafficking from the cell surface, a mechanism proposed in recent studies of psychedelic pharmacology (Vargas et al., 2023).

While these findings provide valuable insight into psychedelics, several questions remain. In many experiments, the neurons were exposed to psilocin for extended periods. In contrast, clinical psychedelic treatments typically involve a single or just a few administrations that can produce long-lasting behavioral effects. This difference is notable because the half-life of psilocybin in the mammalian brain is only about an hour and a half. Future studies could examine whether brief exposures that more closely resemble clinical conditions produce similar effects on neuronal plasticity.

Another important direction will be testing multiple psychedelic compounds and doses. Different psychedelics interact with serotonin and other monoaminergic receptors – as well as their downstream pathways – in distinct ways, potentially influencing their ability to promote plasticity. Finally, emerging research suggests that biological sex may shape neural and behavioral responses to psychedelics and other fast-acting antidepressants (Cohen and Blest-Hopley, 2025). Stem-cell-derived neuronal models could provide a powerful system to investigate how hormones and genetic factors influence these effects in human cells.

Together, the results reported by Schmidt et al. represent an important step toward understanding the mechanisms through which psychedelics reshape human brain cells. By combining stem cell technology with molecular neuroscience tools, researchers are beginning to uncover ways these compounds may help the brain form new connections and potentially inspire new therapies that retain beneficial effects without hallucinogenic properties.
